# Poly(ADP-ribose) polymerase 2 contributes to neuroinflammation and neurological dysfunction in mouse experimental autoimmune encephalomyelitis

**DOI:** 10.1186/1742-2094-10-49

**Published:** 2013-04-22

**Authors:** Amit Kamboj, Ping Lu, Michael B Cossoy, Jillian L Stobart, Brian A Dolhun, Tiina M Kauppinen, Gilbert de Murcia, Christopher M Anderson

**Affiliations:** 1 Department of Pharmacology and Therapeutics, University of Manitoba and Division of Neurodegenerative Disorders, St Boniface Hospital Research, Winnipeg, Canada; 2 Division of Neurodegenerative Disorders, St Boniface Hospital Research, Winnipeg, Canada; 3 Department of Internal Medicine, University of Manitoba, Winnipeg, Canada; 4 Department ‘Intégrité du Génome’ de l’UMR 7175, École Supérieure de Biotechnologie de Strasbourg, Illkirch Cedex, France

**Keywords:** CD4, CD11b, Demyelination, Experimental autoimmune encephalomyelitis, Neuroinflammation, Multiple sclerosis, PARP-1, PARP-2, Th1, Th17

## Abstract

**Background:**

Experimental autoimmune encephalomyelitis (EAE) is an animal model of multiple sclerosis characterized by entry of activated T cells and antigen presenting cells into the central nervous system and subsequent autoimmune destruction of nerve myelin. Previous studies revealed that non-selective inhibition of poly(ADP-ribose) polymerases (PARPs) 1 and 2 protect against neuroinflammation and motor dysfunction associated with EAE, but the role of the PARP-2 isoform has not yet been investigated selectively.

**Results:**

EAE was induced in mice lacking PARP-2, and neurological EAE signs, blood-spine barrier (BSB) permeability, demyelination and inflammatory infiltration were monitored for 35 days after immunization. Mice lacking PARP-2 exhibited significantly reduced overall disease burden and peak neurological dysfunction. PARP-2 deletion also significantly delayed EAE onset and reduced BSB permeability, demyelination and central nervous system (CNS) markers of proinflammatory Th1 and Th17 T helper lymphocytes.

**Conclusions:**

This study represents the first description of a significant role for PARP-2 in neuroinflammation and neurological dysfunction in EAE.

## Introduction

Multiple sclerosis (MS) affects up to 1% of the US population and 2% of Canadians [1,2], and is characterized by central nervous system (CNS) lymphocyte infiltration, autoimmune demyelination, axonal loss and neurodegeneration [3,4]. Experimental allergic encephalomyelitis (EAE) is an animal model that shares many features with human MS, including major histocompatibility complex (MHC)-linked susceptibility, female predominance, paralysis, ataxia, increased cytokine production, and the presence of myelin-reactive T cells [5].

Poly(ADP-ribose) polymerases (PARPs) comprise a superfamily of 18 enzymes involved in DNA repair and genomic stability [6,7]. PARP-1 and PARP-2 share the ability to catalyze poly ADP-ribosylation of target proteins [8] but PARP-1 accounts for about 90% of cellular poly ADP-ribosylation capacity [7,9]. Non-selective PARP-1/2 inhibitors reduce neuroinflammation and neurological dysfunction in rodent EAE [10-12] but it is not yet clear what the relative contributions of PARP-1 and PARP-2 are to this effect. Genetic PARP-1 loss of function in EAE has produced disparate results, both mitigating [13] and enhancing [14] EAE severity in mice. Our aim in the present work was to evaluate the role of PARP-2 deletion in EAE.

## Methods

All animal experiments were performed in accordance with guidelines of and approved by the Institutional Animal Care and Use Committee, University of Manitoba. EAE was induced in 8-week-old *parp-2*^*-/-*^ female mice [9] or wild-type littermate C57Bl/6 controls by immunizing with myelin oligodendrocyte glycoprotein (MOG)_35–55_ and Freund’s complete adjuvant (FCA) [15]. Two subcutaneous injections with 50 μg of MOG_35-55_ emulsified in FCA containing 200 μg of *Mycobacterium tuberculosis* were given on day 0 and 7 days after. Mice also received intraperitoneal pertussis toxin (0.15 μg in 100 μl phosphate-buffered saline (PBS)) on day 0 and day 2. Body weight and neurological score were measured daily. Mice were assigned a cumulative 14-point score [16], assessing function of tail and mobility of each front limb and each hind limb, up to 35 days after the second immunization. For tail function, a score of 0 is asymptomatic, while 1 refers to weakness and 2 is full paralysis. For the hind or forelimbs, each is assessed separately, with a score of 0 for no symptoms, 1 for weakness, 2 for limb dragging with limited movement, and 3 for full paralysis.

Permeability of the blood-spine barrier was determined at initiation of disease signs, as tracked by loss of >1 g of body mass [17], by injecting mice with 100 μl of 10% sodium fluorescein (Na-Fluor, intraperitoneal administration) and monitoring fluorescence in spinal homogenates after 10 minutes.

For histology and immunofluorescence, cervical and thoracic cord segments were snap frozen and sectioned (10 μm) at peak disease scores, determined empirically for each mouse as the fifth day after initial disease signs (score >0). Tissue was stained with hematoxylin and eosin (H&E) to detect CNS inflammatory infiltrates, or solochrome cyanin to assess myelination, and qualitative assessment of each parameter was performed as described previously [16]. All assessments were made by a blinded observer. For immunohistochemistry, primary antibodies were rat anti-CD11b (EMD-Millipore, Billerica, MA, USA), rat anti-CD4 (BD Biosciences, San Diego, CA, USA), rabbit anti-ROR-γT (Abcam, Cambridge, MA, USA) and mouse anti-T-bet (Abcam). Primary antibodies were applied overnight at 4°C. Secondary antibodies were either Alexa Fluor 488 or 560 conjugates (Life Technologies, Carlsbad, CA, USA).

Non-parametric two-tailed Mann–Whitney tests were used to analyze the differences in EAE neurological signs (two groups). Kruskal-Wallis non-parametric analysis of variance (ANOVA) with Dunn’s multiple comparisons test was used for qualitative assessment of clinical scoring with more than two groups. For all other comparisons, one-way ANOVA followed by Student Newman-Keuls multiple comparison test was used.

## Results

### Silencing PARP-2 protects neurological function and reduces blood-spine barrier permeability in mouse EAE

**Figure 1:**
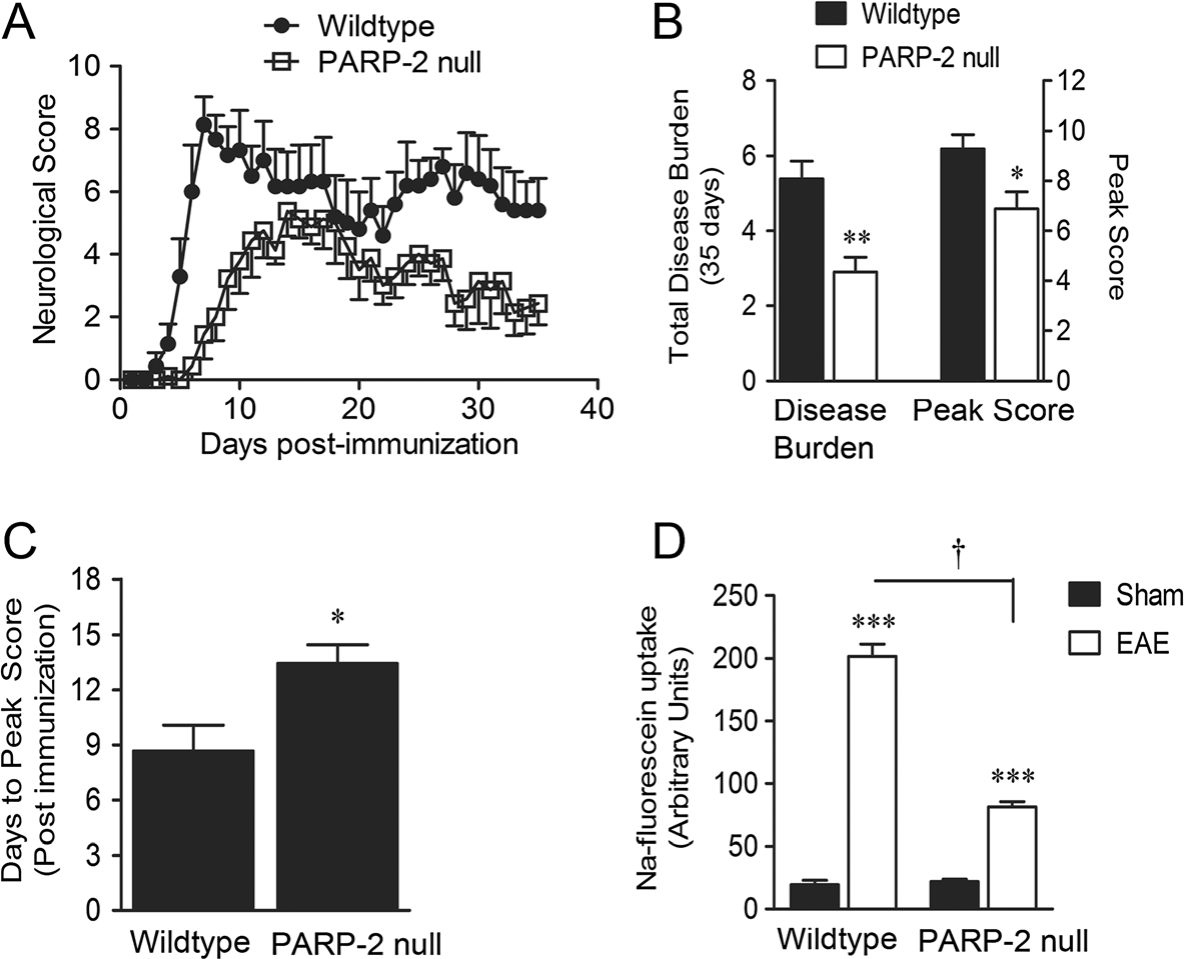
**Deletion of poly(ADP-ribose) polymerase 2 (PARP-2) protects neurological function and blood-spine barrier integrity in experimental autoimmune encephalomyelitis (EAE) mice.** (**A**) PARP-2 null and littermate C57Bl/6 mice were immunized with myelin oligodendrocyte glycoprotein (MOG)_35–55_ and neurological signs of EAE were scored for 35 days. (A) Daily neurological scoring profile for wild-type and PARP-2 null EAE mice. (**B**) Total disease burden (summed score/35 days) and peak neurological score were significantly reduced by PARP-2 deletion. (**C**) The number of days required to reach first peak disease score was significantly increased by PARP-2 deletion. (**D**) Spinal:serum fluorescence was measured following sodium fluorescein (Na-Fluor) administration intraperitoneally just prior to peak disease neurological score. The magnitude of the EAE-induced ratio increase was significantly less in PARP-2 mice than in wild-type animals. Values are expressed as mean ± SEM (n = 6 to 9). (**B**,**C**) *P <0.05, using non-parametric two-tailed Mann–Whitney test. (**D**) ***P <0.001 compared to respective sham control, †*P* <0.001 as indicated using one-way analysis of variance (ANOVA) followed by Student Newman-Keuls multiple comparisons test.

EAE was induced in PARP-2 null mice and wild-type littermate C57Bl/6 controls. Body mass and neurological signs of disease were monitored for 35 days after the second MOG_35-55_ immunization. Figure 1A shows the temporal disease profile for C57Bl/6 (n = 9) and PARP-2 null mice (n = 6). PARP-2 deletion reduced the 35 day total disease burden (aggregate daily score per 35 EAE days) from 4.8 ± 1.5 to 2.6 ± 1.0 and the peak neurological severity score from 9.6 ± 1.1 to 6.9 ± 2.2 (Figure 1B). PARP-2 deletion also significantly increased the number of days required to reach disease peak from 8.7 ± 3.5 days to 13.4 ± 2.7 days after the second immunization (Figure 1C).

Blood-spine barrier (BSB) permeability was assessed immediately following large weight loss (1.89 ± 0.66 g, n = 13) corresponding to peak transient BSB permeability [18]. Following intraperitoneal Na-Fluor administration (10 minutes), spinal fluorescence increased tenfold in EAE mice (n = 4) compared to vehicle-treated (sham, n = 3) controls (Figure 1D). BSB permeability was also significantly enhanced by PARP-2 null EAE mice (n = 4), compared to the PARP-2 null sham group (n = 4), however, the magnitude of this enhancement was considerably smaller (3.7-fold) than observed in wild-type mice.

### PARP-2 deletion reduces spinal inflammatory cell infiltration and demyelination in EAE

**Figure 2:**
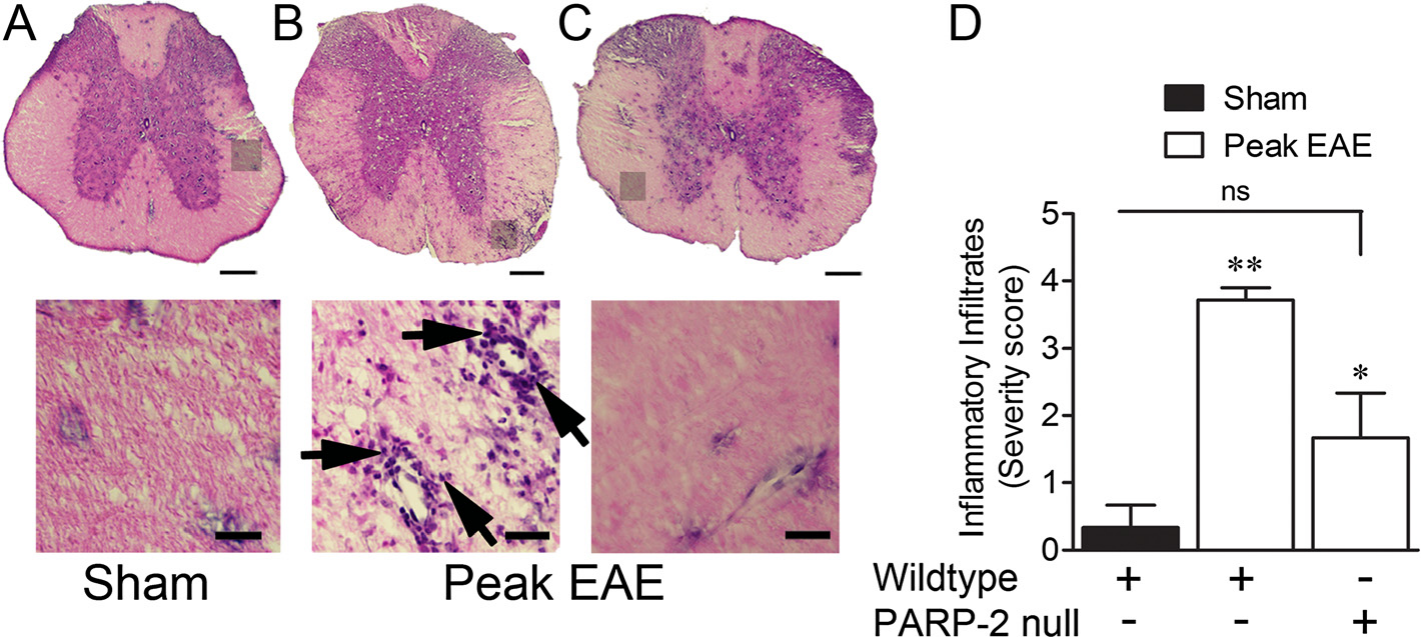
**Poly(ADP-ribose) polymerase 2 (PARP-2) deletion reduces spinal inflammation.** (**A**-**C**) Peak experimental autoimmune encephalomyelitis (EAE) disease ((**B**) wild-type and (**C**) PARP-2 null) and sham control (**A**) spinal cords were subjected to hematoxylin and eosin staining. Wild-type EAE cords have substantial cellular infiltration to the parenchyma (**B**) while the sham control (**A**) and PARP-2 EAE cords (**C**) have minimal visible infiltration. Results are quantified in (**D**). Arrows point to perivascular foci of inflammatory infiltration. Scale bars are 200 μm (top row, (**A**-**C**)) and 25 μm (bottom row, (**A**-**C**)); bottom row in (**A**-**C**) represents magnification of top row framed areas. Values are mean ± SEM (n = 3 to 7). Results in (**D**) were analyzed using Kruskal-Wallis non-parametric analysis of variance (ANOVA) with Dunn’s multiple comparisons test. **P <0.01 compared to sham; *P <0.05 compared to wild-type EAE group; not significant = *P* >0.05.

Mice were sacrificed at peak EAE score, designated as day 5 after each individual animal displayed a neurological score greater than 0, and cervical and thoracic spinal sections were stained with H&E. Wild-type EAE mice exhibited extensive perivascular parenchymal cell infiltration, relative to sham animals (Figure 2A,B), which was reduced by PARP-2 deletion (Figure 2C). Quantification in Figure 2D shows that central infiltration in wild-type mice (n = 7) is statistically reduced by the *parp-2*^*-/-*^ genotype (n = 4).

**Figure 3:**
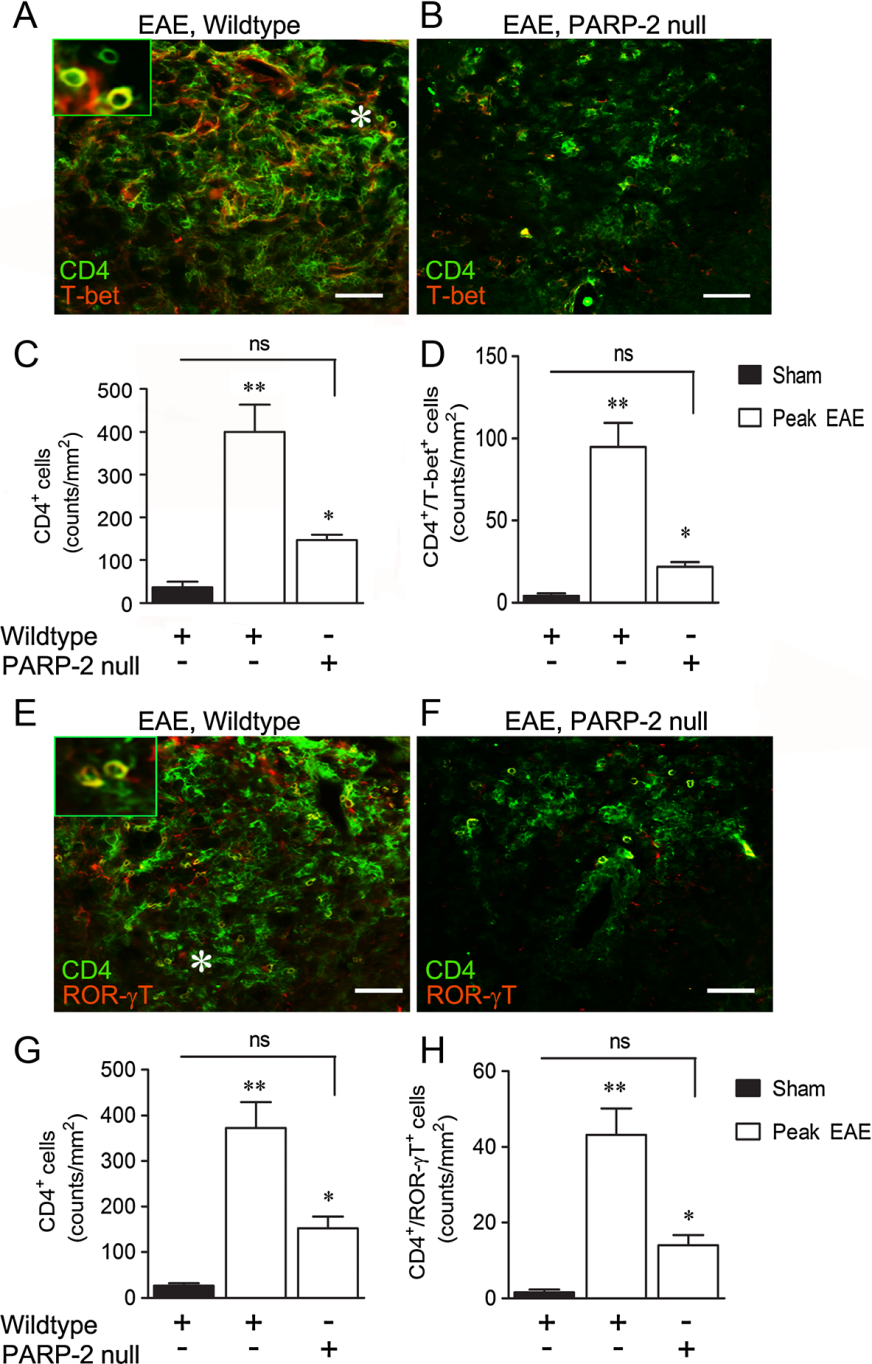
**Poly(ADP-ribose) polymerase 2 (PARP-2) deletion reduces Th1 and Th17 T helper cell infiltration in experimental autoimmune encephalomyelitis (EAE) mice spinal cords.** Sham control and peak EAE spinal cords from wild-type and PARP-2 null mice were subjected to coimmunofluorescence staining using antibodies for CD4 and T-bet (**A**-**D**) or CD4 and ROR-γT (**E**-**H**). Widespread CD4^+^ immunoreactivity (IR) was observed in wild-type EAE spinal sections (**B**), approximately 24% of which was also T-bet^+^. The inset shows a magnified image of the area directly to the right of the asterisk symbol (*) and illustrates cellular colocalization (yellow). (**B**) Representative image of reduced CD4^+^ and CD4^+^/T-bet^+^ infiltration in PARP-2 null EAE cords. (**C**,**D**) Quantifications of CD4^+^ and CD4^+^/T-bet^+^ cell counts and indicate a significant reduction of each with elimination of PARP-2. In (**E**), 12% of CD^+^ cells were also ROR-γT^+^. The inset in (**E**) shows a magnified image of the area directly to the left of the asterisk symbol (*). (**F**) Representative image of reduced CD4^+^ and CD4^+^/ROR-γT^+^ infiltration in PARP-2 null EAE cords. (**G**,**H**) Quantifications of CD4^+^ and CD4^+^/ROR-γT^+^ cell counts and indicate a significant reduction of each with elimination of PARP-2. Results in (**C**,**D**,**G**,**H**) are expressed as means ± SEM and were analyzed using one-way analysis of variance (ANOVA) with the Student Newman-Keuls multiple comparison test. **P <0.01 compared to sham; *P <0.05 compared to wild-type EAE; not significant = *P* >0.05. Scale bars are 25 μm.

**Figure 4:**
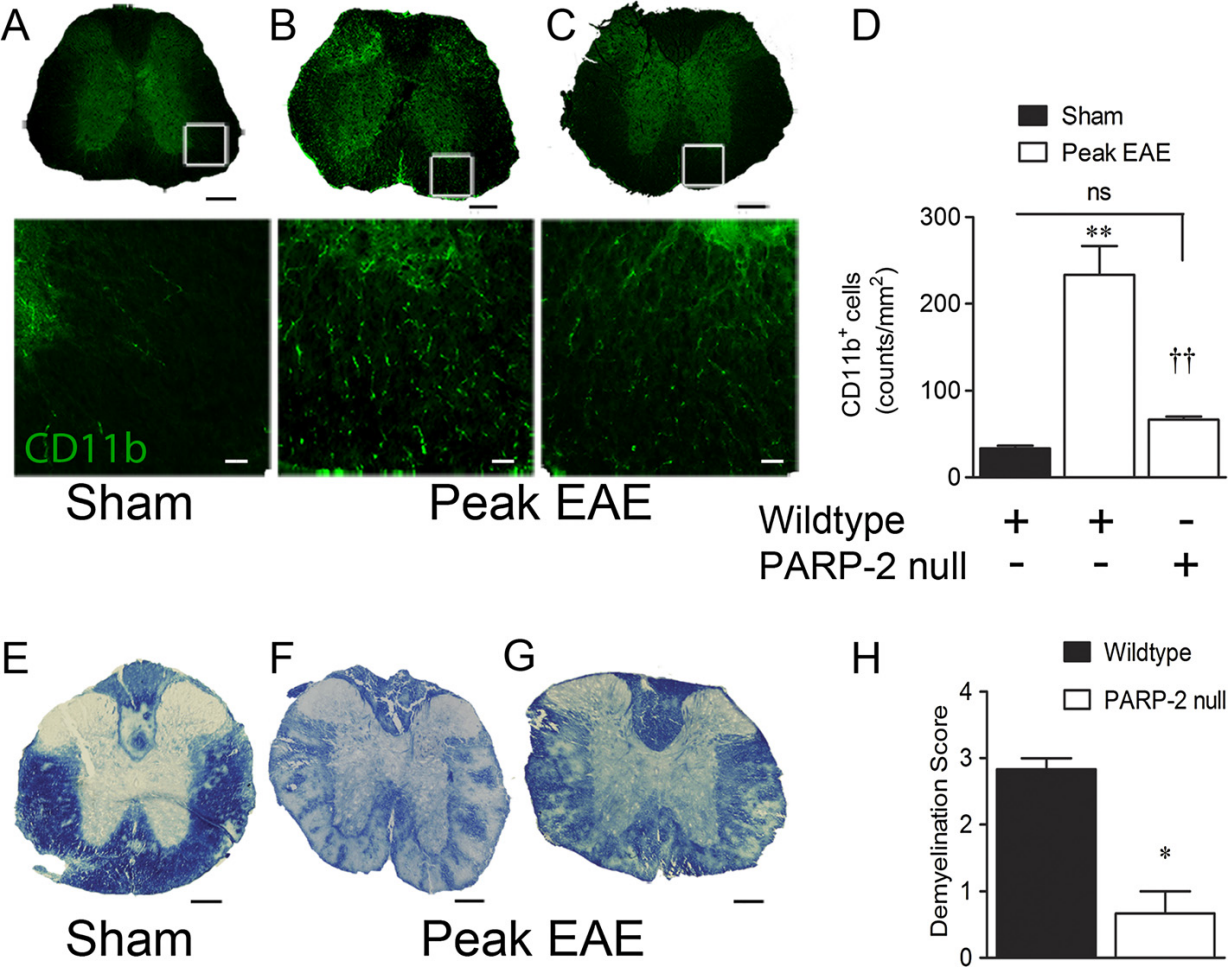
**Poly(ADP-ribose) polymerase 2 (PARP-2) contributes to macrophage accumulation and spinal demyelination in experimental autoimmune encephalomyelitis (EAE).** CD11b immunofluorescence was used to detect macrophage/microglial cells activated by EAE. (**B**) Representative images illustrating elevated CD11b^+^ immunoreactivity in wild-type EAE spinal sections compared with sham sections (**A**). PARP-2 deletion reduced CD11b IR (**C**). Results were quantified in (**D**). Values are mean ± SEM (n = 3 to 7) and were analyzed using one-way analysis of variance (ANOVA) with the Student Newman-Keuls multiple comparison test. **P <0.01 compared to sham; ††*P* <0.01 compared to wild-type EAE; not significant = *P* >0.05. Scale bars are 200 μm (top row, **A**-**C**) and 25 μm (bottom row, (**A**-**C**)). Bottom row represents magnification of framed areas in the top row. Demyelination was assessed in peak disease EAE mice by staining with solochrome cyanin. Wild-type sham spinal cord white matter is thoroughly myelinated, as indicated by deep blue staining (**E**). Wild-type EAE spinal cords exhibit extensive areas of demyelination (**F**). EAE in PARP-2 null spinal cords produced only a low degree of demyelination (**G**) relative to wild-type controls. Results are quantified in (**H**) and show a significant reduction in demyelination of PARP-2 null cords compared to wild-type cords in EAE. Scale bars are 200 μm. Values are mean ± SEM (n = 3 to 6). Results in (**H**) were analyzed using non-parametric two-tailed Mann–Whitney test. *P <0.05 compared to wild-type EAE group.

We next investigated whether there was proinflammatory T lymphocyte infiltration. Parenchymal CD4^+^ cell counts per mm^2^ increased from 36.1 ± 12.8 to 400.1 ± 63.0 in spinal cord sections of EAE mice (n = 7), relative to sham controls (n = 3). PARP-2 deletion significantly reduced EAE CD4^+^ counts to 146.7 ± 12.8 (n = 4; Figure 3A-C). To determine effects of PARP-2 on proinflammatory Th1 and Th17 T helper cell phenotypes, coimmunofluorescence experiments were performed for CD4 and either T-bet (Th1) [19] or ROR-γT (Th17) [20]. EAE increased the number of CD4^+^/T-bet^+^ cells from 3.9 ± 1.6 in sham cords (n = 3) to 94.8 ± 14.6 in EAE (n = 7). Elimination of PARP-2 reduced this to 21.7 ± 2.8 cell/mm^2^ (n = 4; Figure 3D). Similarly, EAE increased CD4^+^/ROR-γT^+^ cell counts from 1.6 ± 0.8 in sham controls to 43.2 ± 6.9 in EAE in a manner dependent on PARP-2, as deletion of PARP-2 reduced EAE counts to 14.0 ± 2.7 (Figure 3E-H). Salutary effects of PARP-2 deletion on spinal microglial/macrophage CD11b accumulation in EAE were also observed. Spinal regions with H&E stain-verified inflammation exhibited widespread CD11b^+^ immunoreactivity (IR) in EAE (Figure 4A, B) that was PARP-2 dependent (Figure 4C, D).

Multifocal areas of reduced solochrome cyanin intensity revealed widespread, diffuse demyelination in wild-type EAE mice (Figure 4E, F) that was significantly reduced in PARP-2 null EAE mice (Figure 4G, H). These results support a role for PARP-2 in EAE-induced spinal demyelination in mice.

## Discussion

We addressed the role of PARP-2 in EAE using *parp-2*^*-/-*^ mice. Embryonic deletion of PARP-2 protected EAE mice from neuroinflammation and neurological dysfunction. Overall, *parp-2*^*-/-*^ mice had significantly lower total disease burden over the 35 day period studied, compared to wild-type C57Bl/6 controls. Peak EAE neurological scores were also significantly reduced by eliminating PARP-2. In practical terms, this peak protection means that hindlimb paralysis and tail/forelimb weakness observed in wild-type EAE mice were prevented in *parp-2*^*-/-*^ mice, which experienced average maximal scores corresponding to hindlimb weakness but no paralysis or tail and forelimb effects. In addition, while we found a discernable peak EAE score in *parp-2*^*-/-*^ mice, the peak was shifted temporally further away from EAE induction, compared to wild-type mice, representing a 50% delay in EAE peak effect. We also evaluated BSB permeability and series of markers for EAE neuroinflammation. The *parp-2*^*-/-*^ genotype significantly reduced EAE-induced BSB permeability and spinal distributions of CD4^+^/T-bet^+^, CD4^+^/ROR-γT^+^, and CD11b^+^ inflammatory infiltrates, as well as demyelination in the cervical and thoracic spinal cord. Taken together, these data demonstrate that embryonic deletion of PARP-2 protects nerve myelin and motor performance while reducing neuroinflammation associated with EAE.

Our observation that PARP-2 deletion is protective in EAE raises the possibility that PARP inhibitors shown previously to have beneficial effects in EAE are effective at least partially by interfering with the PARP-2 isoform. In agreement, PJ34 [11], 6(5H)-phenanthridinone (PHE) [10] and 5-aminoisoquinolinone (5-AIQ) [13] improved EAE outcomes and all compete for the NAD^+^ binding site of both PARP-1 and PARP-2 [21-23], and we (data not shown) and others [14] found that PARP-1 deletion is not protective but rather enhances peak disease severity. However, PARP-1 deletion reduced neuroinflammation and improved EAE outcome in another EAE model [13], making categorical conclusions about the relative roles of PARP-1 and PARP-2 in EAE difficult at this point. It should be noted that embryonic deletion of PARP isoforms prior to EAE could have different effects on EAE outcomes than adult pharmacological or genetic loss of function. For example, *parp-1*^*-/-*^ mice have higher numbers of regulatory and EAE-activated CD4^+^/CD8^+^ T lymphocytes compared with wild-type animals [14,24] and *parp-2*^*-/-*^ mice exhibit reduced thymus size, cellularity and numbers of peripheral T cell precursor thymocytes [25]. In both of these cases, there could be a phenotypic predisposition to a different immune response prior to EAE induction that would not be present in wild-type animals. Further work employing models in which PARP isoforms can be selectively inhibited or deleted just prior to or during EAE (conditional knockouts) are required to precisely delineate the neuroinflammatory roles of PARP-1 and PARP-2, and to determine the potential usefulness of these isoforms as therapeutic targets in adult EAE.

## Conclusions

The current study implicates PARP-2 in neuroinflammation and neurological signs of EAE for the first time. Identification of a selective role for PARP-2 in EAE progression establishes a novel therapeutic target of interest for neuroinflammation and MS.

## Competing interests

The authors report no conflicts of interest.

## Authors’ contributions

AK was responsible for inducing EAE, animal care, keeping record of daily weights, neurological scoring of the EAE animals, manuscript preparation and performing assessments of blood-spine barrier permeability, histology, and statistical analysis. PL carried out immunohistochemical analysis. MBC prepared tissues for histology and immunohistochemistry. JLS assisted AK in preparing myelin oligodendrocyte glycoprotein, inducing EAE and in transcardiac perfusion. BAD performed the preliminary EAE experiments and was involved in conceptual design. TMK contributed significantly to the intellectual development of the project. GM provided the PARP-2 knockout strain for performing EAE studies. CMA conceived and designed the study, supervised all personnel and helped to draft the manuscript. All authors read and approved the final manuscript.
